# Adenosine A1 receptors of the medullary solitary tract arbitrate the nicotine counteraction of neuroinflammation and cardiovascular dysfunction in septic rats

**DOI:** 10.1038/s41598-023-44601-w

**Published:** 2023-10-19

**Authors:** Amany E. El-Naggar, Mai M. Helmy, Sahar M. El-Gowilly, Mahmoud M. El-Mas

**Affiliations:** 1 Department of Pharmacology and Toxicology, Faculty of Pharmacy, Alexandria University, Alazarita, Alexandria, 21521 Egypt; 2 Department of Pharmacology and Toxicology, College of Medicine, Kuwait University, Kuwait City, Kuwait

**Keywords:** Infection, Inflammation, Receptor pharmacology, Cardiovascular biology, Experimental models of disease

## Abstract

The cholinergic pathway plays a crucial role in improving inflammatory end-organ damage. Given the interplay between cholinergic and adenosinergic neurotransmission, we tested the hypothesis that central adenosine A1 receptors (A1ARs) modulate the nicotine counteraction of cardiovascular and inflammatory insults induced by sepsis in rats. Sepsis was induced by cecal ligation and puncture (CLP) 24-h before cardiovascular measurements. Nicotine (25–100 µg/kg i.v.) dose-dependently reversed septic manifestations of hypotension and impaired heart rate variability (HRV) and cardiac sympathovagal balance. Like nicotine, intracisternal (i.c.) administration of N(6)-cyclopentyladenosine (CPA, A1AR agonist) to CLP rats increased indices of HRV and sympathovagal balance. Moreover, greater surges in these parameters were noted upon simultaneous nicotine/CPA administration. The favorable influences of nicotine on blood pressure and HRV in sepsis were diminished after central blockade of A1ARs by i.c. 8-Cyclopentyl-1,3-dipropylxanthine (DPCPX). Molecular studies revealed that (i) septic rises in myocardial and brainstem nucleus of solitary tract (NTS) NFκB expression were abrogated by nicotine and largely reinstated after blockade of A1ARs, and (ii) A1AR expression in the same areas was reduced by DPCPX. It is concluded that myocardial and medullary A1ARs facilitate the cholinergic counteraction of cardiac and neuroinflammation induced by sepsis and interrelated cardiomyopathic and neuropathic hitches.

## Introduction

Sepsis is a life-threatening hyperinflammatory condition resulting from severe systemic infection^1^. Despite advances in the management of sepsis, it remains a leading cause of disability and mortality in intensive care units^2^. Neuroinflammation and myocardial and autonomic dysfunction are major adverse events of sepsis that have been attributed to the overproduction of proinflammatory cytokines^3–6^. By contrast, The cholinergic antiinflammatory pathway is a key mechanism that acts reflexly to suppress the inflammatory response induced by sepsis^7^. A number of studies have demonstrated that nicotine and nicotinic agonists inhibit the synthesis and release of pro-inflammatory cytokines in endotoxemia and sepsis^8–10^. We have previously reported that nicotine alleviates cardiovascular and autonomic derangements and associated brainstem inflammation in lipopolysaccharides (LPS)-induced endotoxemia^11,12^.

The adenosine A1 receptor (A1AR) is a subset of a cluster of receptors that are activated by the endogenous immunomodulator adenosine. A1ARs are highly expressed in central neurons, astrocytes and microglia^13^ and involved in the regulation of neuroinflammation^14,15^. In periphery, high levels of A1ARs are also found in heart, inflammatory cells and adipose tissue^16^. There is a discrepancy regarding the role of A1ARs in inflammatory conditions. While a proinflammatory role for A1ARs has been described in some experimental settings such as acute pancreatitis, sepsis and ischemia reperfusion injury of lung, heart and liver, others suggest an antiinflammatory role for A1ARs in renal ischemia–reperfusion injury, experimental allergic encephalomyelitis and sepsis^17^. Moreover, evidence of a functional interplay exists between cholinergic and adenosinergic neurotransmissions in the central nervous system, but it focuses mainly on A_2A_ARs and their interaction with behavioral, antinociceptive, and arterial baroreceptor depressant actions of nicotine^18–20^.

To our knowledge, little or no information is available in the literature on the specific role of A1ARs in cardiovascular and autonomic sequels of sepsis and its interaction with the cholinergic anti-inflammatory pathway. The present study opted to evaluate whether the reversal by nicotine of cardiovascular, autonomic, and inflammatory aberrations induced by sepsis are modulated by central A1ARs. The experimental approach adopted here involved the investigation of the effect of separate or combined treatment with nicotine or pharmacologic ligands of A1ARs on septic manifestations of hypotension and defective heart rate variability (HRV) in the septic model of cecal ligation and puncture (CLP) in rats. Additionally, the molecular underpinning of these interactions was determined by assessing the expression of A1ARs and nuclear factor kappa B (NFκB) inflammatory signal in the myocardium as well as in cardiovascular-sensitive neuronal circuits of the brainstem. Experiments were undertaken in conscious freely moving rats pre-instrumented with indwelling femoral and intracisternal catheters.

## Results

### CLP causes hypotension and cardiac autonomic dysfunction

Baseline values of hemodynamic and cardiac autonomic functions of sham and CLP rats measured after hemodynamic stabilization and prior to any drug administration are shown in Table 1. Compared with sham rats, CLP rats showed a significant decrease in blood pressure (BP) that amounted approximately to 22 mmHg (116.2 ± 4.8 vs. 95.7 ± 3.5 mmHg) whereas heart rate (HR) was not significantly altered. The fall in BP was paralleled with significant reductions in time-domain (standard deviation of NN intervals, SDNN) and frequency-domain parameters (total power and low frequency power LFnu). The LF/HF ratio was significantly lower in CLP compared with sham rats by about 65%, indicating a shift in the sympathovagal balance towards parasympathetic predominance.

**Table 1:** Baseline values of MAP, HR and HRV indices measured 24 h after CLP or sham operation.

Parameter	Sham	CLP
MAP, mmHg	116.2 ± 4.8	95.7 ± 3.5*
HR, beats/min	383.7 ± 19.2	407.4 ± 22.1
SDNN, msec	4.62 ± 0.40	3.13 ± 0.32*
Total power, msec^2^	22.49 ± 2.63	6.09 ± 0.65*
LFnu	12.39 ± 1.44	5.43 ± 1.01*
HFnu	81.47 ± 3.72	64.01 ± 13.39
LF/HF	0.17 ± 0.02	0.06 ± 0.01*

Values are means ± SEM of 8 observations. The unpaired Student’s *t* test was employed to test for statistical significance.

*MAP* mean arterial pressure, *HR* heart rate, *SDNN* standard deviation of NN intervals, *LFnu* power in low frequency range (normalized unit), *HFnu* power in high frequency range (normalized unit).

*P < 0.05 vs. corresponding sham values.

### Nicotine reverses septic manifestations of hypotension and cardiac autonomic depression

**Figure 1:**
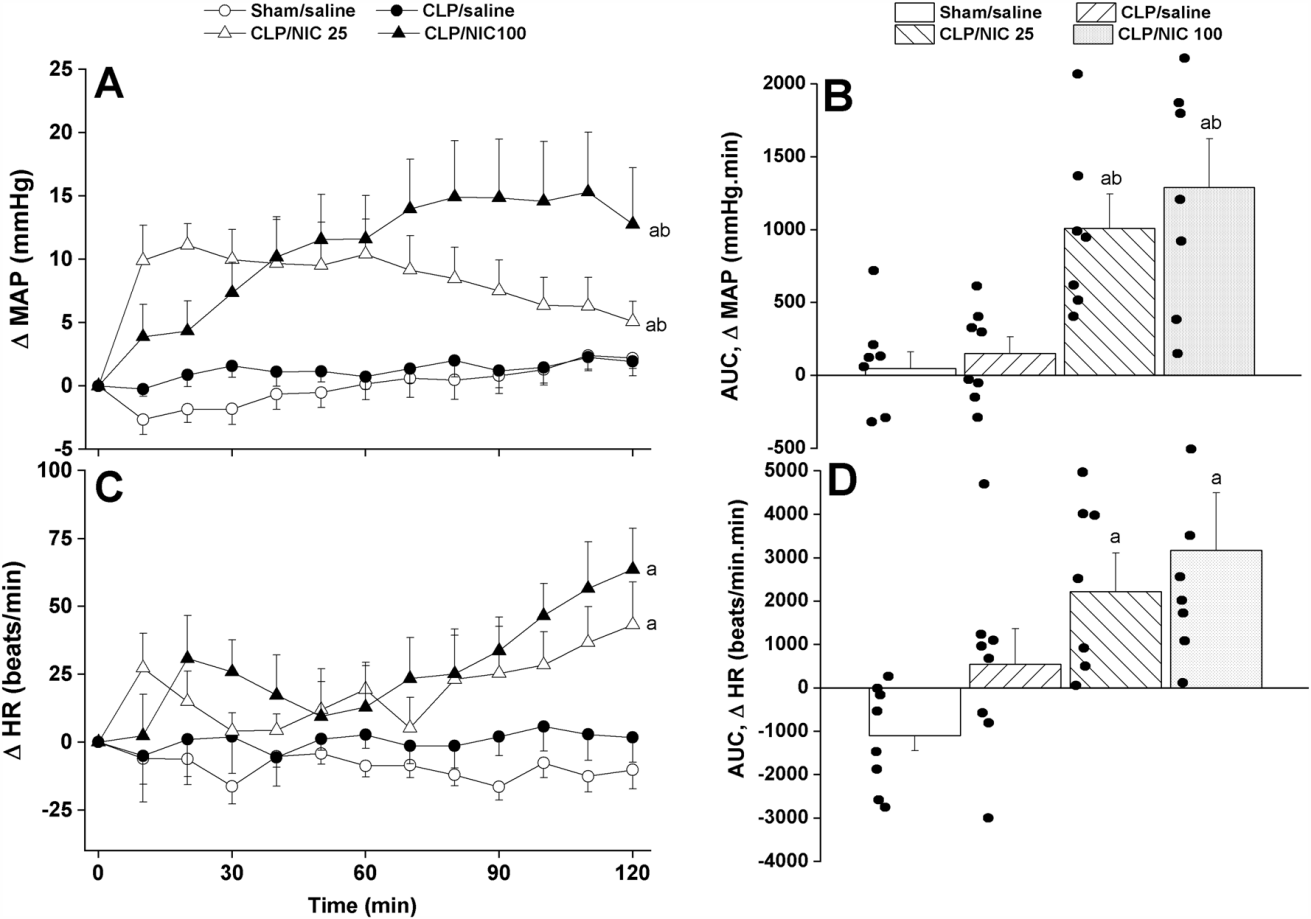
The dose-dependent increase in time-course (panels **A**–**C**) and cumulative changes (areas under the curves, AUCs, panels (**B**–**D**)) in mean arterial pressure (MAP) and heart rate (HR) caused by i.v. nicotine (25 or 100 μg/kg) in septic (cecal ligation and puncture, CLP) male rats. Values are means ± SEM of 7–8 observations. The one-way (panels **A**–**C**) and repeated measures ANOVA (panels **B**–**D**) followed by the Tukey’s post hoc test were employed to test for statistical significance. ^a^P < 0.05 vs. “sham/saline”, ^b^P < 0.05 vs. “CLP/saline”.

**Figure 2:**
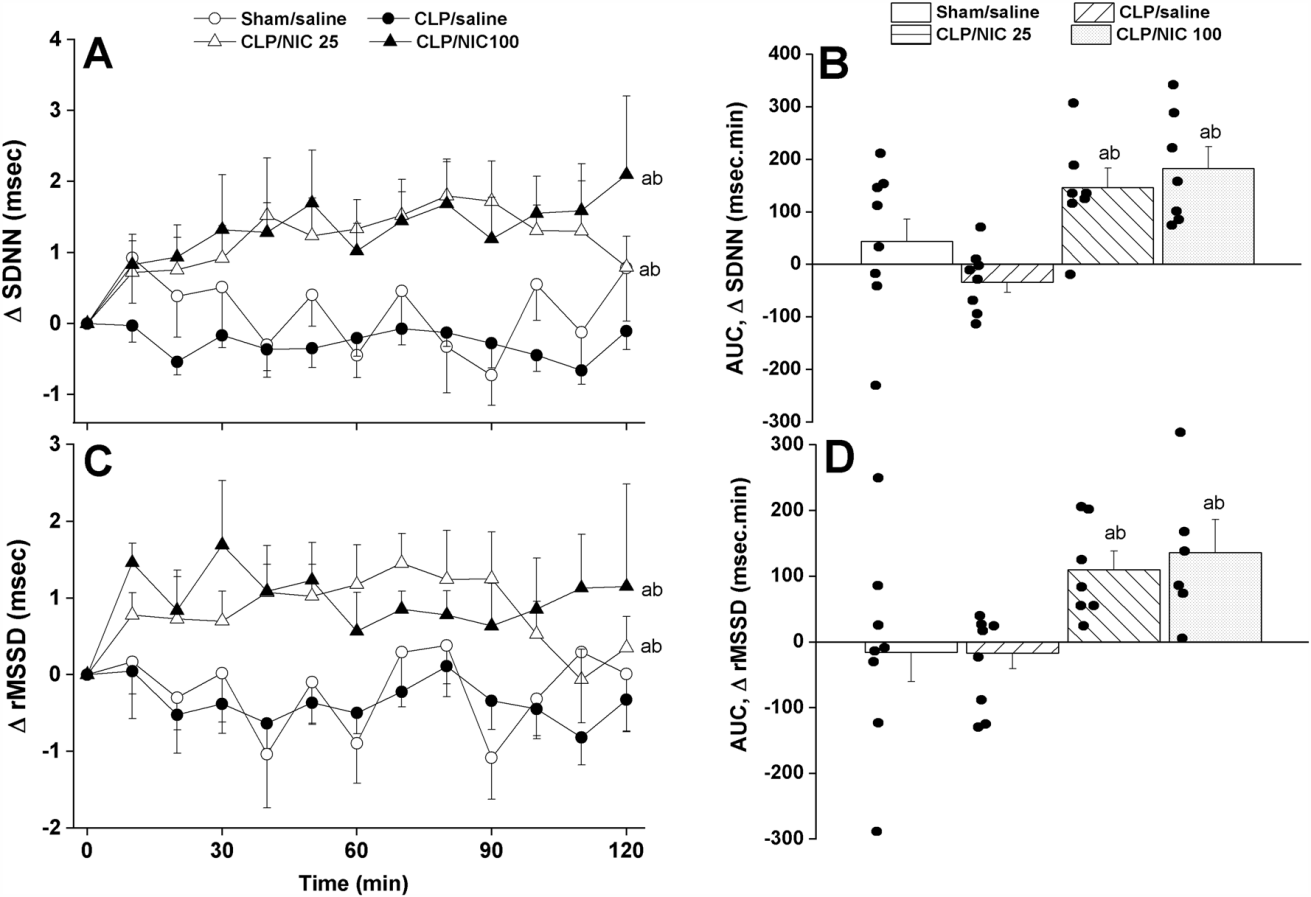
The dose-dependent increase in time-course (panels **A**–**C**) and cumulative changes (areas under the curves, AUCs, panels **B**–**D**) in time-domain indices of HRV (SDNN, the standard deviation of NN intervals, and rMSSD, the square root of the mean squared differences of successive NN intervals, as measures of overall HRV and parasympathetic activity, respectively) evoked by i.v. nicotine (25, 100 μg/kg) in septic (cecal ligation and puncture, CLP) male rats. Values are means ± SEM of 6–8 observations. The one-way (panels **A**–**C**) and repeated measures ANOVA (panels **B**–**D**) followed by the Tukey’s post hoc test were employed to test for statistical significance. ^a^P < 0.05 vs. “sham/saline”, ^b^P < 0.05 vs. “CLP/saline”.

**Figure 3:**
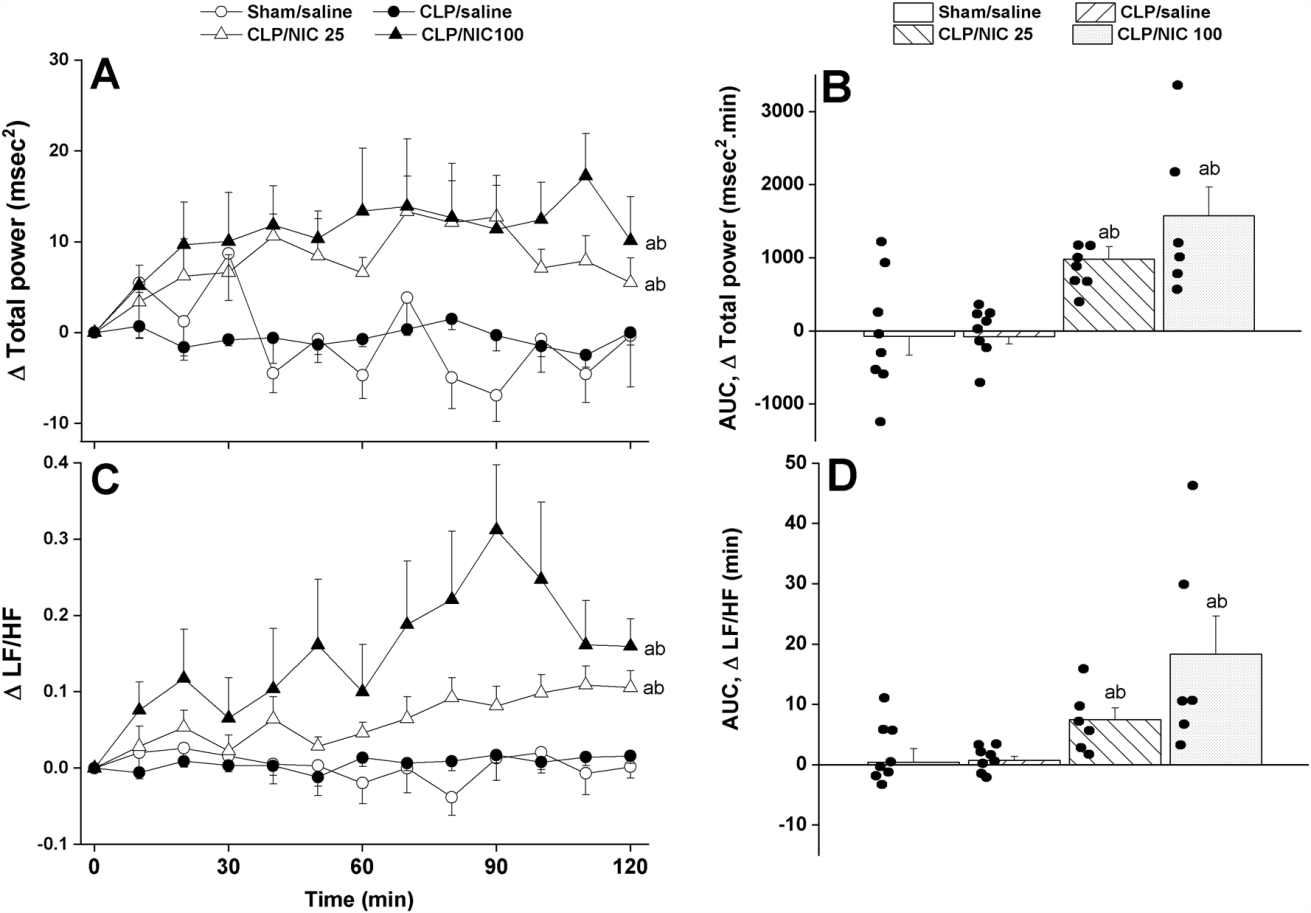
The dose-dependent increase in time-course (panels **A**–**C**) and cumulative changes (areas under the curves, AUCs, panels **B**–**D**) in frequency-domain indices of HRV (total power and LF/HF ratio, the ratio of low frequency range to high frequency range as measures of overall HRV and sympathovagal balance, respectively) induced by i.v. nicotine (25, 100 μg/kg) in septic (cecal ligation and puncture, CLP) male rats. Values are means ± SEM of 6–8 observations. The one-way (panels **A**–**C**) and repeated measures ANOVA (panels **B**–**D**) followed by the Tukey’s post hoc test were employed to test for statistical significance. ^a^P < 0.05 vs. “sham/saline”, ^b^P < 0.05 vs. “CLP/saline”.

The time-course and cumulative effects (area under the curves, AUCs) of i.v. administration of nicotine (25, 100 µg/kg) are depicted in Figs. 1, 2 and 3. In contrast to no effect for saline, the treatment of CLP rats with nicotine significantly and dose-dependently elevated mean arterial pressure (MAP) during the entire 2-h period of the experiment (Fig. 1A,B). The pressor response elicited by nicotine appeared to have overturned the fall in MAP observed initially in CLP rats prior to nicotine administration (see baseline values in Table 1). Remarkably, the comparison of MAP values seen in nicotine (100 µg/kg)-treated CLP rats and in saline-treated sham rats by the end of the 2-h observation period revealed no statistically significant differences (115.3 ± 4.1 and 113.9 ± 5.4 mmHg, respectively). The treatment of CLP with nicotine also caused significant rises in HR compared with respective values in saline-treated rats (Fig. 1C,D).

Analysis of HRV in the time and spectral domains demonstrated that the sepsis-related disturbances in cardiac autonomic control observed 24 h after CLP were improved after i.v. nicotine administration. The time-domain indices of the cardiac autonomic activity (SDNN, Fig. 2A,B) and vagal cardiotonic activity (the square root of the mean squared differences of successive NN intervals, rMSSD, Fig. 2C,D) were both significantly increased in nicotine-treated CLP rats. In similar fashions, spectral FFT measures of cardiac autonomic control demonstrated that nicotine boosted the total power, which reflect the overall oscillations of the R-R intervals or total cardiac autonomic control (Fig. 3A,B), as well as LF/HF ratios (a predictor of sympathovagal balance, Fig. 3C,D). The data imply the ability of nicotine to bring the defective cardiac sympathovagal balance in septic rats back to its normal levels featured in control rats.

### Central A1ARs facilitate the nicotine counteraction of septic manifestations

**Figure 4:**
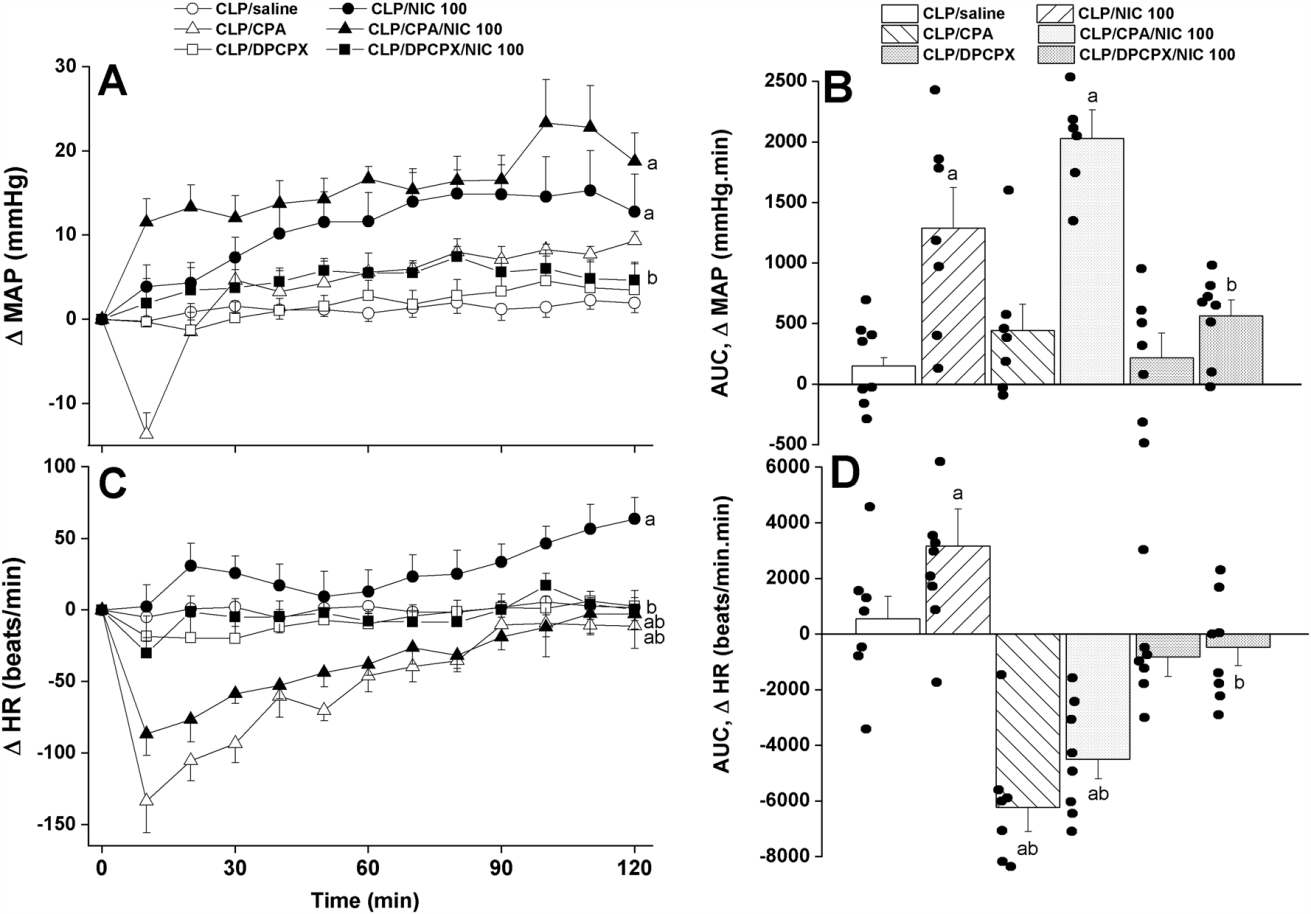
Effects of central adenosine A1 receptor activation (CPA) or blockade (DPCPX) on the nicotine-evoked changes in time-course (panels **A**–**C**) and cumulative (areas under the curves, AUCs, panels **B**–**D**) values of mean arterial pressure (MAP) and heart rate (HR) in septic (cecal ligation and puncture, CLP) male rats. Values are means ± SEM of 6–8 observations. The one-way (panels **A**–**C**) and repeated measures ANOVA (panels **B**–**D**) followed by the Tukey’s post hoc test were employed to test for statistical significance. ^a^P < 0.05 vs. “CLP/saline”, ^b^P < 0.05 vs. “CLP/NIC”.

**Figure 5:**
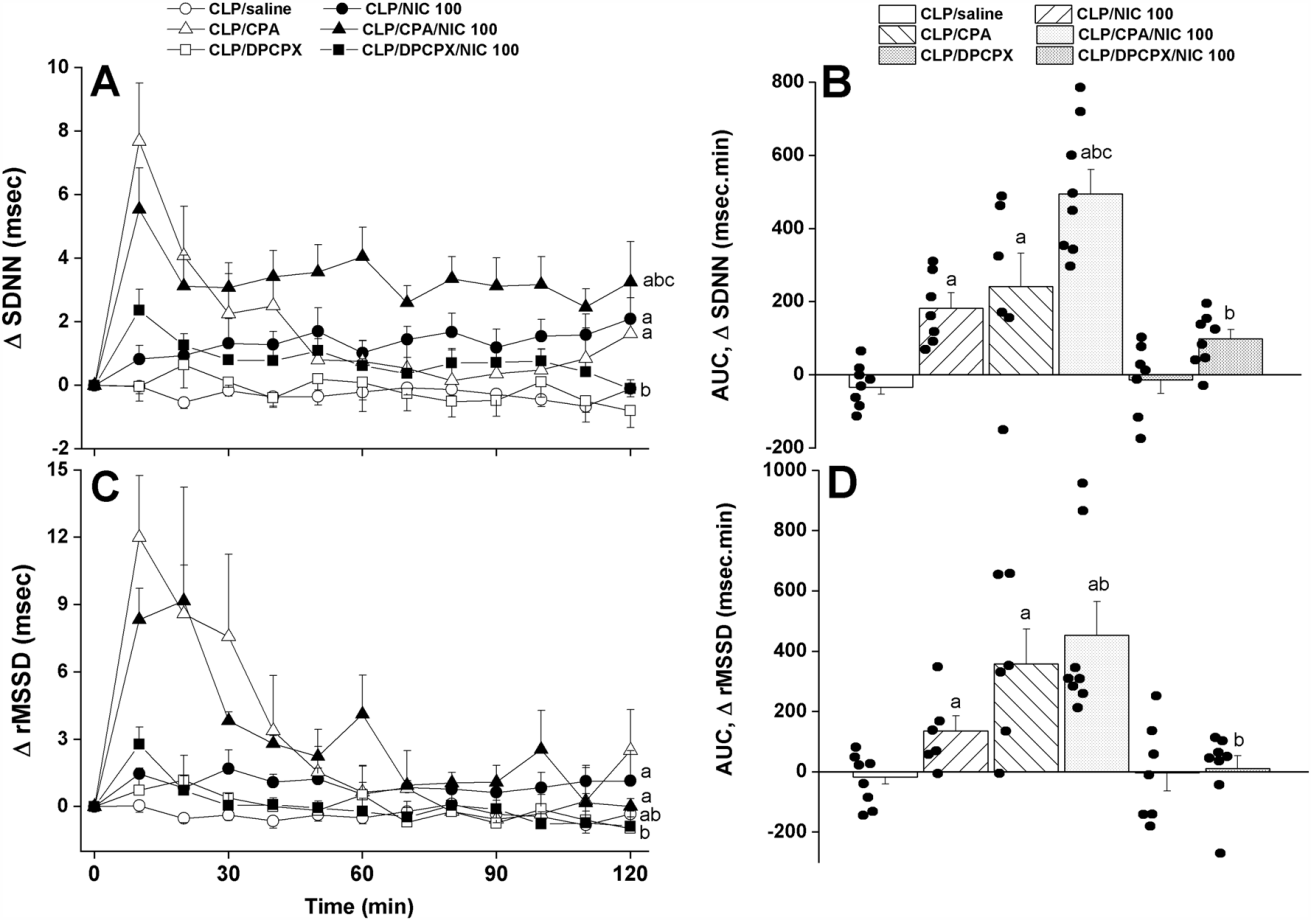
Effects of central adenosine A1 receptor activation (CPA) or blockade (DPCPX) on the nicotine-evoked increase in time-course (panels **A**–**C**) and cumulative changes (areas under the curves, AUCs, panels **B**–**D**) in time-domain indices of HRV (SDNN, the standard deviation of NN intervals, and rMSSD, the square root of the mean squared differences of successive NN intervals, as measures of overall HRV and parasympathetic activity, respectively) in septic (cecal ligation and puncture, CLP) male rats. Values are means ± SEM of 6–8 observations. The one-way (panels **A**–**C**) and repeated measures ANOVA (panels **B**–**D**) followed by the Tukey’s post hoc test were employed to test for statistical significance. ^a^P < 0.05 vs. “CLP/saline”, ^b^P < 0.05 vs. “CLP/NIC”, ^c^P < 0.05 vs. “CLP/CPA”.

**Figure 6:**
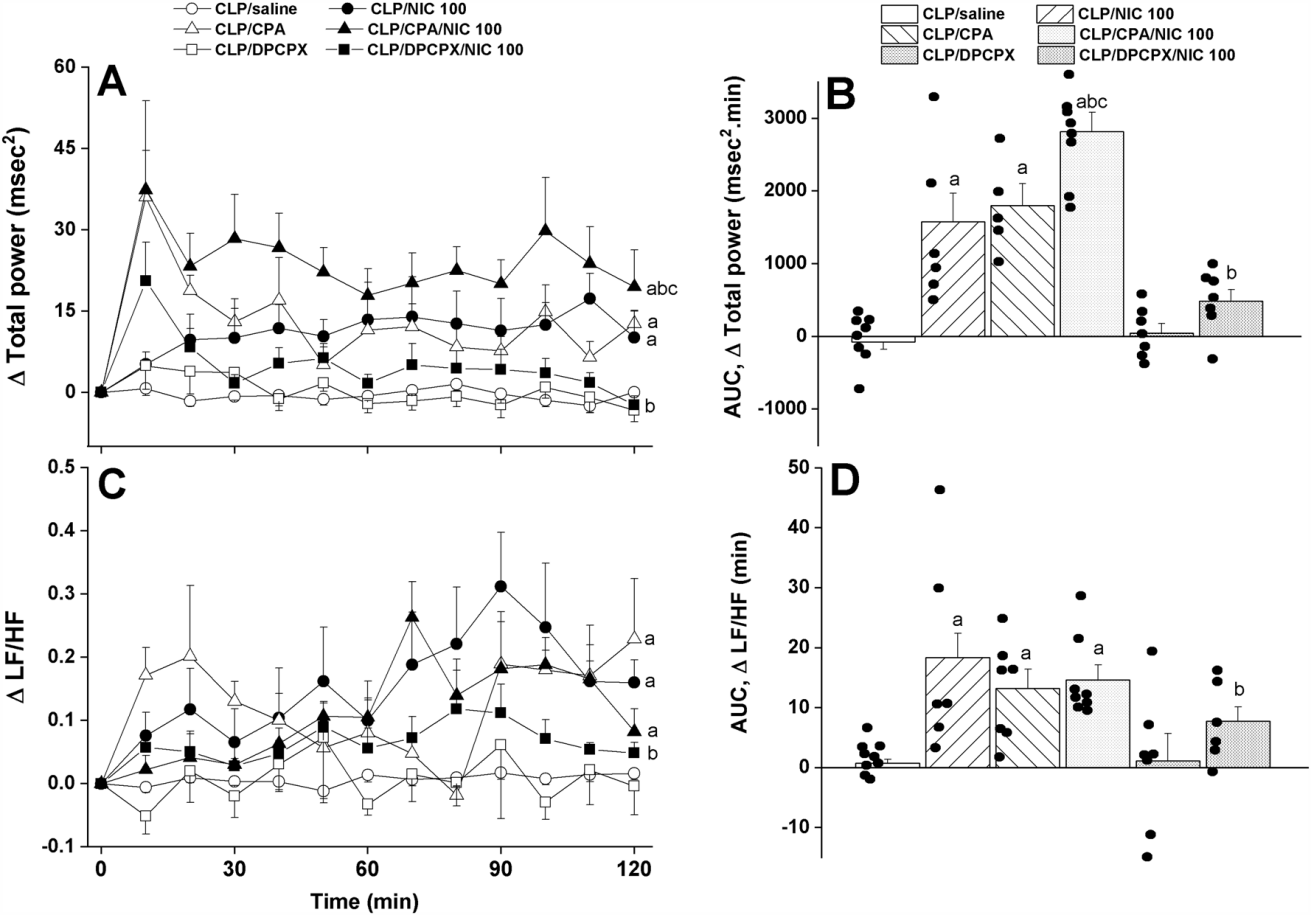
Effects of central adenosine A1 receptor activation (CPA) or blockade (DPCPX) on the nicotine-evoked increase in time-course (panels **A**–**C**) and cumulative changes (areas under the curves, AUCs, panels **B**–**D**) in frequency-domain indices of HRV (total power and LF/HF ratio, ratio of low frequency range to high frequency range, as measures of overall HRV and sympathovagal balance, respectively) in septic (cecal ligation and puncture, CLP) male rats. Values are means ± SEM of 6–8 observations. The one-way (panels **A**–**C**) and repeated measures ANOVA (panels **B**–**D**) followed by the Tukey’s post hoc test were employed to test for statistical significance. ^a^P < 0.05 vs. “CLP/saline”, ^b^P < 0.05 vs. “CLP/NIC”, ^c^P < 0.05 vs. “CLP/CPA”.

Figures 4, 5 and 6 illustrate the time-course and cumulative effects (AUCs) of central activation or blockade of A1ARs on the nicotine-mediated counteraction of septic responses. The i.c. administration of CPA (A1AR agonist, 4 µg/rat) prior to nicotine (100 µg/kg) had no effect on the nicotine-evoked pressor response but reversed the concomitant tachycardia (Fig. 4). When given alone, the same CPA regimen significantly reduced the HR but failed to alter MAP. On the other hand, the blockade of central A1ARs by i.c. DPCPX (6 µg/rat) caused no hemodynamic changes on its own but virtually abolished the pressor and tachycardic responses elicited by subsequently administered nicotine (Fig. 4).

By analogy, the data showed that the A1AR agonist CPA favorably impacted the impaired HRV when used alone and accentuated the nicotine responses in septic rats. Both time (SDNN, Fig. 5A,B) and spectral measures (total power, Fig. 6A,B) of total cardiac autonomic activity were comparably increased by individual treatments with nicotine and CPA and significantly greater rises in these parameters were noted in septic rats treated simultaneously with the combined therapies. On the other hand, the LF/HF ratio, cardiac sympathovagal balance, was increased by nicotine or CPA, but no further potentiation when the combined regimen was adopted (Fig. 6C,D). In all circumstances, the antagonist studies demonstrated that the promising HRV responses evoked by nicotine were significantly suppressed in septic rats after pharmacological elimination of A1ARs by DPCPX (Figs. 5, 6). Together, the data are consistent with a prominent role for intact and functional A1ARs in evoking the cholinergically-mediated rectification of cardiovascular profile in septic rats.

### Effects on cardiac and neuronal expression of NFκB and A1ARs

**Figure 7:**
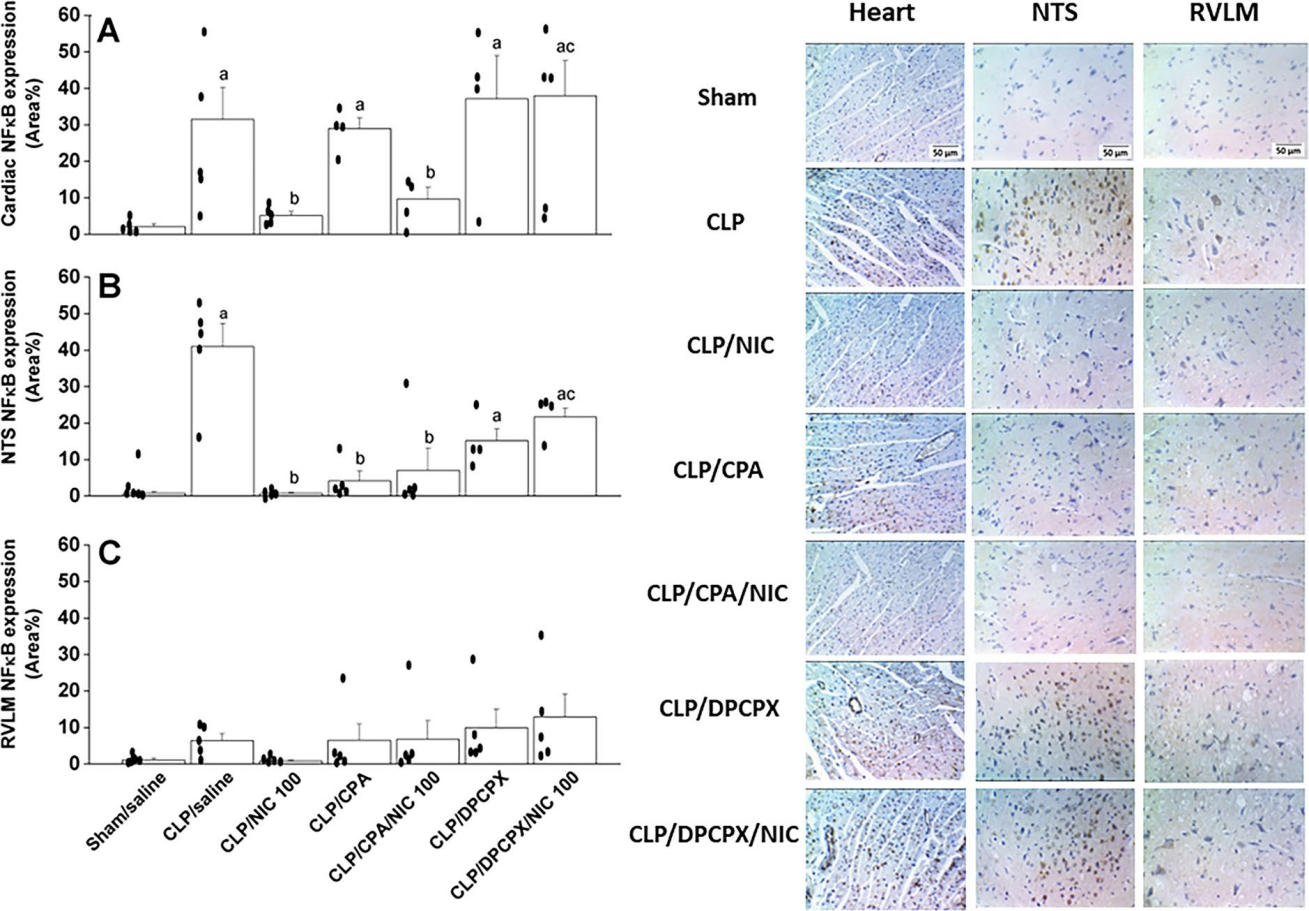
Effects of i.v. nicotine and/or i.c. CPA (A1R agonist) or DPCPX (A1R antagonist) on NFκB expression in hearts and brainstem areas of NTS (nucleus tractus solitarius) and RVLM (rostral ventrolateral medulla) of septic (cecal ligated and puncture, CLP) rats. Values are means ± SEM of 4–5 observations. The one-way ANOVA followed by the Tukey’s post hoc test was employed to test for statistical significance. ^a^P < 0.05 vs. “sham/saline”, ^b^P < 0.05 vs. “CLP/saline”, ^c^P < 0.05 vs. “CLP/NIC”.

Immunohistochemical studies showed that the effects of nicotine and/or A1AR ligands on NFκB and A1ARs expression in the heart and brainstem of septic rats depended on the types of tissue and neuronal site. The expression of proinflammatory cytokine NFκB was significantly increased in the myocardium and medullary nuclei of the solitary tract (NTS) (Fig. 7A,B). The heightened NFκB expression in response to the septic challenge was eliminated after systemic administration of the 100 μg/kg dose of nicotine. Further, the depressant action of nicotine on the inflammatory response was maintained and reversed following i.c. treatment with the A1AR agonist (CPA) and antagonist (DPCPX), respectively (Fig. 7A,B). Unlike the heart and NTS, NFκB expression in the rostral ventrolateral medulla (RVLM) was not influenced by the septic insult or any of the pharmacologic interventions employed (Fig. 7C).

**Figure 8:**
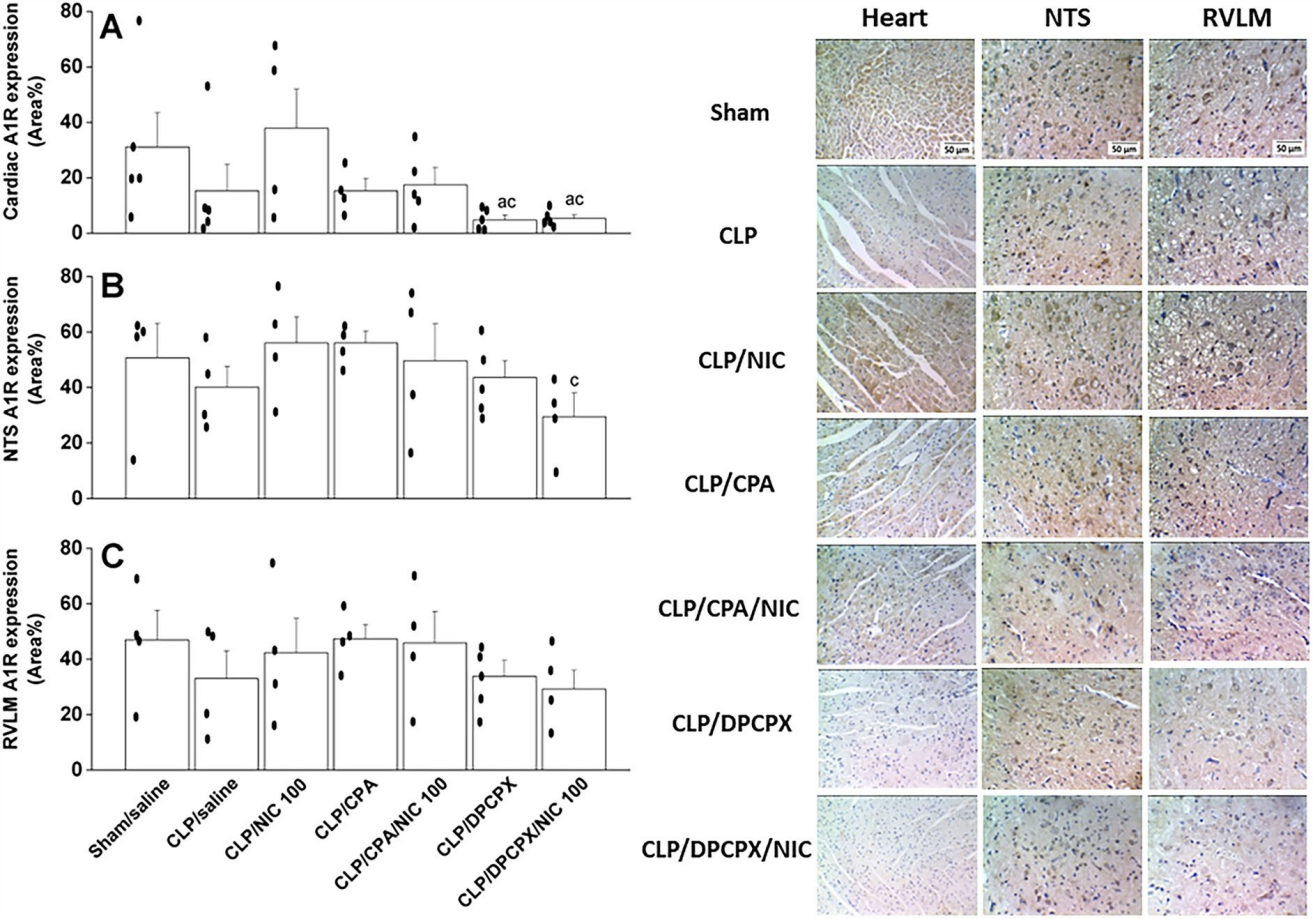
Effects of i.v. nicotine and/or i.c. CPA (A1R agonist) or DPCPX (A1R antagonist) on A1AR expression in hearts and brainstem areas of NTS (nucleus tractus solitarius) and RVLM (rostral ventrolateral medulla) of septic (cecal ligated and puncture, CLP) rats. Values are means ± SEM of 4–5 observations. The one-way ANOVA followed by the Tukey’s post hoc test was employed to test for statistical significance. ^a^P < 0.05 vs. “sham/saline”, ^c^P < 0.05 vs. “CLP/NIC”.

Immunohistochemical staining of A1ARs showed that CLP caused no significant change in A1AR expression in heart or brainstem areas of NTS and RVLM (Fig. 8). Moreover, while A1AR activation by CPA failed to alter A1AR expression in all tissues, the blockade of A1ARs by DPCPX significantly reduced A1AR staining in the heart as well as in NTS neurons of nicotine-treated septic rats (Fig. 8). Representative images depicting the NFκB and A1AR staining in cardiac and medullary sites are also shown in Figs. 7 and 8, respectively.

## Discussion

The current study is the first to report on the role of central A1ARs in the cholinergic modulation of cardiovascular irregularities evoked by sepsis. We found that systemically administered nicotine dose-dependently reversed the hypotension response to sepsis and normalized the concomitant disturbances in HRV and sympathovagal balance. Pharmacological targeting of central A1ARs showed that the favorable effects of nicotine were (i) preserved or possibly augmented following i.c. administration of the selective A1AR agonist CPA, (ii) ameliorated after central blockade of A1ARs by DPCPX. Molecularly, DPCPX noticeably reduced the protein expression of A1ARs and mitigated the downregulatory effect of nicotine on the heightened inflammatory response (NFκB expression) in peripheral (heart) and brainstem (NTS) sites. The data imply a pivotal role for neuronal circuits of medullary A1ARs in the processing of the cholinergically-mediated counteraction of sepsis-related neuroinflammatory and cardiovascular assault.

In a previous experimental study, we reported a shielding effect for nicotine against hypotension and cardiac autonomic neuropathy elicited by sepsis^12^. In this latter study, however, exogenously administered LPS was used to simulate the effect of sepsis. The LPS model has traditionally been criticized as being an imperfect model of sepsis because it is characterized by high, rapid and transient elevation of cytokines, which differs qualitatively and quantitatively from the pattern of inflammatory response often associating human sepsis^21^. Moreover, the LPS insult is mediated mainly via the toll-like receptor 4-dependent inflammatory cascade, which does not accurately reflect the multifaceted cytokine storm in human sepsis^22^. To circumvent this limitation, the current study employed the CLP rat model which is believed to optimally replicate features and complexity of human sepsis^21,23^. The current study, therefore, is the first to investigate the dose-related effects of nicotine on cardiovascular and inflammatory sequelae of sepsis in the CLP rat model.

The analysis of HRV in the time- and frequency domains is a powerful tool that is routinely used to measure cardiac autonomic activity. Remarkably, the impairment of HRV is believed to be associated with increased risks of cardiovascular morbidity and sudden cardiac death in sepsis^11,12,24^ as well as in other pathological conditions such as diabetes^25^ and heart failure^26^. In the present study, evidence of cardiac autonomic neuropathy in the CLP rat model was verified by the (i) diminished overall HRV as reflected by the significant reductions in time (SDNN) and spectral (total power) heart dynamics, and (ii) parasympathetic predominance indexed by the decline in the cardiac sympathovagal balance (LF/HF ratio). The latter change in sympathovagal activity appears to have been mediated via the reduction in cardiac sympathetic activity (LF oscillations) as no significant changes have been noted in parasympathetic activity (HF oscillations) (see Table 1). These spectral data are consistent with reports of impaired cardiac sympathetic modulation observed in patients with early onset sepsis^24,27–29^. Further, our observations that disturbances in the cardiac autonomic profile and concomitant falls in blood pressure, distinct hallmarks of sepsis and septic shock^3,4,30^, were attenuated following systemic administration of nicotine in a dose-related fashion are in compliance with the defensive role of the nicotinic cholinergic pathway in sepsis^7,11,12^.

Considering the widespread distribution of A1ARs in cardiac and central sites and their role in the regulation of neuroinflammation^13–16,31^, we asked if central A1ARs constitute an intermediary element in neuronal circuits involved in the cholinergic modulation of sepsis. In support of this postulate, pharmacological studies of the current study demonstrated that the capacity of nicotine to reverse the hypotensive and cardiac autonomic responses evoked by sepsis was fully abolished or at least compromised after central blockade of A1ARs by i.c. DPCPX. More proof for the potential rectifying action of A1ARs in the setting of sepsis emerged from agonistic studies in which the activation of central A1ARs by CPA favorably impacted the deteriorated HRV profile in septic rats and this effect was more manifest in septic rats treated consecutively with CPA plus nicotine. It is tempting to speculate that the presence of functional central A1ARs is a prerequisite for the recruitment of the defensive central cholinergic machinery against cardiovascular and autonomic manifestations of sepsis. While the current study is the first to report on the facilitatory interaction between cholinergic and adenosinergic A1AR pathways in sepsis, a similar positive role for A2aARs in behavioral^20,32^ and antinociceptive effect of nicotine^18^ have been previously established. That said, it should be noted that more studies are necessary to further consolidate the role of cholinergic/A1AR interaction in septic cardiac autonomic disruption. This can possibly be accomplished by assessing the sympathetic efferent discharge (e.g. cardiac, renal) as well as arterial baroreflex function. These suggestions form a framework for future studies in our laboratory.

The molecular inflammatory basis of the cholinergic/adenosinergic interaction in sepsis was assessed by quantifying the protein expression of the proinflammatory cytokine NFκB, a prime transcription factor that triggers a complex array of septic inflammatory mediators^33^, in the heart and brainstem nuclei of the NTS and RVLM. The latter neuronal pools were particularly targeted because they are critically implicated in cardiovascular control and considerably endowed with large populations of A1ARs and nicotinic cholinergic receptors^34–36^. Also, the NTS and its polysynaptic connections with medullary and hypothalamic centers are critical for the central processing of peripheral inflammatory signals^12,37^. We found that the tremendous rises evoked by sepsis in cardiac/NTS NFκB expression were virtually abolished by nicotine and resurfaced upon central blockade of A1ARs by DPCPX. The lack of similar neuroinflammatory responses in the RVLM suggest the involvement of RVLM-independent circuits, e.g. forebrain, dorsal motor nucleus of the vagus, and hypothalamic paraventricular nucleus^37,38^, in the post-NTS processing of the inflammatory signal in the central nervous system. Together, the evidence obtained from molecular studies reinforces the role of inflammatory signals in peripheral (heart) and central (NTS) tissues in the cholinergic modulation of inflammatory and cardiovascular consequences of sepsis.

Unlike NFκB, immunohistochemical data revealed no changes in cardiac or neuronal expression of A1ARs in septic rats, thereby precluding a possible role for A1ARs in the adverse cardiovascular effects caused by sepsis. Reported studies on the effects of sepsis on A1AR abundance were conflicting and largely depended on the specific model of sepsis employed, tissue type, and insult duration. In one study, Rogachev et al.^39^ reported that E-coli-induced peritonitis resulted in a counterregulatory elevation in A1AR levels on peritoneal mesothelial cells and leukocytes that peaked 12 h after E-coli inoculation and returned to basal levels at 24 h. To the contrary, others demonstrated a reduced abundance of A1ARs on lymphocytes following LPS administration^40^. Notably, despite the lack of a change in A1AR expression in our current sepsis model, A1AR blockade by DPCPX caused a clear decline in A1AR expression in hearts and NTS neurons obtained from nicotine-treated septic rats (Fig. 8A,B). Considering the repressing effect of DPCPX on the nicotine counteraction of cardiovascular signs of sepsis, it is conceivable that the physiologic A1AR expression acts tonically to disclose the privileged nicotine effects.

It is important to comment on the machinery by which peripheral and central pathways interact to mediate neuroinflammatory responses associated with sepsis. Evidence suggests that peripherally released inflammatory mediators can signal the brain via humoral and neural pathways. The humoral pathways include the passage of cytokines through the circumventricular organs that lack blood brain barrier or via binding to cytokine receptors expressed on cerebral endothelial cells^41^. Additionally, sepsis is often associated with disruption of the blood brain barrier, which facilitates the entry of immune cells into the brain, activates glial cells and consequently amplifies the inflammatory signal^42,43^. Interestingly, Pavlov et al. reviewed that the vagus afferent sensory fibers in the nodose ganglion terminate primarily within medullary areas of the NTS, RVLM, and dorsal motor nucleus of the vagus. The latter serves as the principal neuroanatomical site of origin of preganglionic vagus efferent fibers^37^. Moreover, neuronal pools of the same medullary areas play key roles in the processing of peripheral inflammatory signals as well as in cardiovascular homeostasis^12,35,37^.

In conclusion, the current study provides novel pharmacologic and molecular data that implicate neuronal circuits of A1ARs in the nucleus of the solitary in uncovering the cholinergic defense against neuroinflammatory and consequent cardiovascular insults induced by the septic challenge. The study highlights a therapeutic potential for selectively targeting A1ARs in sepsis. More experimental and clinical studies are warranted to verify this assumption.

## Materials and methods

### Animals

Adult male Wistar rats (200–250 g, Faculty of Pharmacy animal facility, Alexandria University, Alexandria, Egypt) were used. All experimental protocols and animal manipulations were approved by the Institutional Animal Care and Use Committee, Alexandria University, Egypt (Approval No. AU/06.2020.6.7.2.73) and were in accordance with the ARRIVE guidelines (https://arriveguidelines.org).

### Drugs

Betadine (povidone iodine solution 10%), heparin sodium (5000 I.U/mL), pencitard (1,200,000 I.U benzathine benzyl penicillin), thiopental (500 mg thiopental sodium), N(6)-cyclopentyladenosine (CPA), 8-Cyclopentyl-1,3-dipropylxanthine (DPCPX) (Sigma Chemical Co., St. Louis, MO, U.S.A.), nicotine (Merck Schuchardt OHG, Hohenbrunn, Germany). CPA and DPCPX were dissolved in DMSO.

### Induction of sepsis by cecal ligation and puncture (CLP)

CLP was performed as previously described^44^ one day before conducting cardiovascular studies. The abdominal area of thiopental-anesthetized rats (50 mg/kg, i.p.) rats was shaved and disinfected by betadine. A midline laparotomy, about 1.5 cm, was performed, and the cecum was exposed and one third of the distal end was tightly ligated. The cecum was then punctured three times on the same side using a 21-gauge needle and gently compressed to extrude a small amount of fecal content. Thereafter, the cecum was returned to the abdominal cavity and the abdominal musculature and skin were stitched.

### Intracisternal cannulation (i.c.)

A stainless steel guide cannula (23 G, Miami, FL, USA) was implanted into the cisterna magna under thiopental anesthesia (50 mg/kg, i.p.) 5 days before the day of experiment (i.e. 4 days before intravascular cannulation and CLP) as described in our previous studies^45,46^. The guide cannula was passed between the occipital bone and the cerebellum so that its tip protruded into the cisterna magna. The cannula was secured in place with dental luting cement (Glass Ionomer, Hangzhou, China). Each rat received an i.m. injection of benzathine benzyl penicillin (60,000 U) and was housed individually.

### Intravascular cannulation

Intravascular cannulation was performed as previously described^47,48^ on the same day of CLP. Briefly, rats were anesthetized with thiopental (50 mg/kg, i.p.) and polyethylene catheters were inserted in the abdominal aorta and vena cava via the femoral artery and vein for blood pressure (BP) measurement and i.v. drug administration, respectively. Catheters were tunneled subcutaneously, exteriorized at the back of the neck between the scapulae, flushed with heparin (100 U/ml), and plugged by stainless steel pins. Experiments started one day later to ensure full recovery of rats. On the day of experiment, the arterial catheter was connected to a BP transducer (model P23XL; Astro-Med, West Warwick, RI) that was attached through MLAC11 Grass adapter cable to a computerized data acquisition system with LabChart-7 pro software (Power Lab 4/35, model ML866/P; AD Instruments Pty Ltd., Castle Hill, Australia) for the measurement of BP, heart rate (HR) and HRV as detailed below.

### Time-domain analysis of HRV

Two time-domain parameters of the cardiac autonomic activity were measured: SDNN, the standard deviation of NN intervals (R-R interval of normal beats) and rMSSD, the square root of the mean squared differences of successive NN intervals^49,50^. The NN intervals were computed from the HR (i.e. the reciprocal of HR in ms). SDNN is a measure of the overall activity of the autonomic nervous system and correlates with the total power (variance of NN intervals)^50^. rMSSD is a measure of the parasympathetic activity and correlates with the high frequency (HF) power of the spectrum^50,51^. SDNN and rMSSD were measured before (baseline) and at 10 min intervals after drug treatments.

### Frequency-domain analysis of HRV

Spectral hemodynamic fluctuations, quantitative indices of cardiac autonomic control, were used to reflect changes in sympathetic and parasympathetic activity. Frequency-domain analysis of HRV were analyzed based on the fast Fourier transform algorithm (FFT)^50,52,53^. Spectra were integrated into 2 specific frequency bands, low-frequency (LF, 0.25–0.75 Hz) and high-frequency (HF, 0.75–3 Hz) bands and expressed in normalized units (LFnu and HFnu). The LF/HF ratio is taken as a measure of the cardiac sympathovagal balance. Spectral data were estimated before (baseline) and at 10 min intervals after drug treatments.

### Immunohistochemistry

The technique described in previous studies^54,55^ was employed for immunohistochemical determination of the protein expression of NFκB and A1ARs. Rat hearts and brainstems were fixed in 10% formaldehyde solution and embedded in paraffin blocks. Approximately 5 μm sections of rat heart and brainstem (− 12.0 mm relative to bregma)^47,56^ were cut and placed on positively charged adhesion glass slides (Epredia™, Braunschweig, Germany), then deparaffinized in xylene and rehydrated in a series of decreasing ethanol concentrations (100, 95 and 70%). Heat-induced epitope retrieval was done by immersing slides in coplin jars containing 10 mM citrate buffer solution and incubated in a microwave at power 100 for 1 min then power 30 for 9 min. Endogenous peroxidases were blocked by 3% hydrogen peroxide for 10 min. The primary polyclonal antibodies (rabbit anti-NFκB p65, Bioss ™, USA) and (rabbit anti-A1AR, ThermoFisher, USA) were diluted (1:300) as instructed by the manufacturer, applied to the slides and then sections were incubated at 4 °C overnight. The secondary antibody (HRP conjugate) was applied for 30 min. The chromogen 3,3′-diaminobenzidine (DAB) was prepared and applied as instructed by the manufacturer for protein visualization. Slides were counterstained with hematoxylin and dipped in ascending concentrations of alcohol and then xylene. Images were taken by OptikamB9 digital camera (Optika® microscopes, Italy) and Fiji Image J software version 1.51n (National Institutes of Health, Bethesda, Maryland, USA) was employed to measure the area fraction of DAB positive staining in heart and brainstem areas of nucleus tractus solitarius (NTS) and rostral ventrolateral medulla (RVLM).

### Protocols and experimental design

#### Dose-dependent effects of nicotine on inflammatory and cardiovascular responses to CLP-induced sepsis

**Figure 9:**
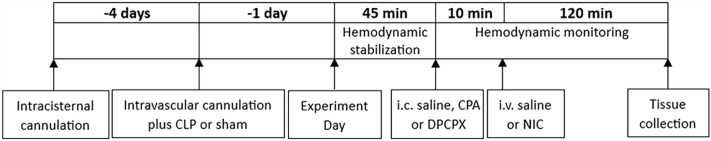
Diagrammatic representation of the timeline of surgical procedures and drug regimens.

The objective of this experiment was to test the effect of systemic nicotine treatment on inflammatory and cardiovascular manifestations of CLP-induced sepsis. Male Wistar rats were randomly assigned to four treatment groups (n = 8 each): (i) sham/saline, (ii) CLP/saline, (iii) CLP/nicotine (25 μg/kg), or (iv) CLP/nicotine (100 μg/kg)^11^. Saline or nicotine was administered intravenously after a stabilization period of at least 45 min, and hemodynamic monitoring continued for 2 h thereafter. Changes in MAP, HR, and time (SDNN, rMSSD) and frequency (total power, LF 0.25–0.75 Hz; HF 0.75–3 Hz, LF/HF ratio) domain indices of HRV were computed at 10 min intervals. At the end of hemodynamic monitoring, rats were euthanized with an overdose of thiopental (100 mg/kg), hearts and brainstems were quickly removed, fixed in 10% formaldehyde solution, then processed for immunohistochemical measurement of the protein expression of NFκB and A1ARs as described earlier. The diagrammatic representation of the timelines of surgical procedures and drug regimens employed in the experiments is depicted in Fig. 9.

#### Central A1AR modulation of the nicotine counteraction of septic manifestations

This experiment investigated the effect of central A1AR agonism or antagonism on cardiac dysfunction and neuroinflammation induced by sepsis in the absence and presence of nicotine. Four more groups of conscious rats (n = 8 each) subjected to CLP operation randomly received one of the following regimens: (i) i.c. CPA (A1AR agonist, 4 µg/rat)^57^ plus i.v. saline, (ii) i.c. CPA plus i.v. nicotine (100 μg/kg), (iii) i.c. DPCPX (A1AR antagonist, 6 µg/rat)^19^ plus i.v. saline, or (iv) i.c. DPCPX plus i.v. nicotine (100 μg/kg). A 10-min interval was allowed between the two successive treatments of each regimen, and hemodynamic monitoring continued for 2 h after the last treatment. Changes in MAP, HR, and HRV parameters were computed at 10 min intervals. At the end of hemodynamic monitoring, rats were euthanized with an overdose of thiopental (100 mg/kg), hearts and brainstems were quickly removed, fixed in 10% formaldehyde solution, and processed for immunohistochemical measurement of the protein expression of NFκB and A1ARs as described earlier. The diagrammatic representation of the timeline of surgical procedures and drug regimens employed in the experiments is depicted in Fig. 9.

### Statistical analysis

Values are expressed as means ± S.E.M. The area under the curves (AUCs) were calculated for individual parameters as a measure of the cumulative drug effect over the entire experiment period. The AUC was computed by GraphPad Prism 8.0.2. using trapezoidal integration and zero line as the baseline^58^. The settings were adjusted to consider the peaks that go above and below the baseline and the “net area” was computed by subtracting the area of peaks below the baseline from the area of peaks above the baseline. The Shapiro–Wilk test was applied to check for normal distribution of data using GraphPad Prism 8.0.2. The unpaired Student’s *t* test was used to compare the means of two independent groups. The one-way or repeated measures ANOVA followed by the Tukey’s post hoc test was used for multiple comparisons. These analyses were performed by GraphPad InStat, software release 3.05. Probability levels less than 0.05 were considered significant.

### Supplementary Information

Supplementary Information 1.

Supplementary Information 2.

Supplementary Information 3.

Supplementary Information 4.

Supplementary Information 5.

Supplementary Information 6.

## Data Availability

Raw data are provided as Supplementary Data.

## References

[1] Singer M (2016). The third international consensus definitions for sepsis and septic shock (sepsis-3). JAMA.

[2] Fleischmann-Struzek C (2020). Incidence and mortality of hospital- and ICU-treated sepsis: Results from an updated and expanded systematic review and meta-analysis. Intensive Care Med..

[3] Pancoto JA, Corrêa PB, Oliveira-Pelegrin GR, Rocha MJ (2008). Autonomic dysfunction in experimental sepsis induced by cecal ligation and puncture. Auton. Neurosci..

[4] Rudiger A, Singer M (2013). The heart in sepsis: From basic mechanisms to clinical management. Curr. Vasc. Pharmacol..

[5] Sallam MY, El-Gowilly SM, Abdel-Galil AG, El-Mas MM (2016). Central GABAA receptors are involved in inflammatory and cardiovascular consequences of endotoxemia in conscious rats. Naunyn Schmiedebergs Arch. Pharmacol..

[6] Moraes CA, Zaverucha-do-Valle C, Fleurance R (2021). Neuroinflammation in sepsis: Molecular pathways of microglia activation. Pharmaceuticals (Basel).

[7] Wang DW, Yin YM, Yao YM (2016). Vagal modulation of the inflammatory response in sepsis. Int. Rev. Immunol..

[8] Kojima H, Ito K, Tsubone H, Kuwahara M (2011). Nicotine treatment reduces LPS-induced sickness responses in telemetry monitoring rats. J. Neuroimmunol..

[9] Wang H (2004). Cholinergic agonists inhibit HMGB1 release and improve survival in experimental sepsis. Nat. Med..

[10] Pavlov VA (2007). Selective alpha7-nicotinic acetylcholine receptor agonist GTS-21 improves survival in murine endotoxemia and severe sepsis. Crit. Care Med..

[11] Sallam MY, El-Gowilly SM, El-Gowelli HM, El-Lakany MA, El-Mas MM (2018). Additive counteraction by α7 and α4β2-nAChRs of the hypotension and cardiac sympathovagal imbalance evoked by endotoxemia in male rats. Eur. J. Pharmacol..

[12] Sallam MY, El-Gowilly SM, Fouda MA, Abd-Alhaseeb MM, El-Mas MM (2019). Brainstem cholinergic pathways diminish cardiovascular and neuroinflammatory actions of endotoxemia in rats: Role of NFκB/α7/α4β2AChRs signaling. Neuropharmacology.

[13] Sheth S, Brito R, Mukherjea D, Rybak LP, Ramkumar V (2014). Adenosine receptors: Expression, function and regulation. Int. J. Mol. Sci..

[14] Tsutsui S (2004). A1 adenosine receptor upregulation and activation attenuates neuroinflammation and demyelination in a model of multiple sclerosis. J. Neurosci..

[15] Liu G (2018). Adenosine binds predominantly to adenosine receptor A1 subtype in astrocytes and mediates an immunosuppressive effect. Brain Res..

[16] Olah ME, Stiles GL (1995). Adenosine receptor subtypes: Characterization and therapeutic regulation. Annu. Rev. Pharmacol. Toxicol..

[17] Blackburn MR, Vance CO, Morschl E, Wilson CN, Wilson CN, Mustafa SJ (2009). Adenosine receptors in health and disease. Handbook of Experimental Pharmacology.

[18] Homayounfar H, Jamali-Raeufy N, Sahebgharani M, Zarrindast MR (2005). Adenosine receptor mediates nicotine-induced antinociception in formalin test. Pharmacol. Res..

[19] El-Mas MM, El-Gowilly SM, Fouda MA, Saad EI (2011). Role of adenosine A2A receptor signaling in the nicotine-evoked attenuation of reflex cardiac sympathetic control. Toxicol. Appl. Pharmacol..

[20] Gill WD, Shelton HW, Burgess KC, Brown RW (2020). Effects of an adenosine A(2A) agonist on the rewarding associative properties of nicotine and neural plasticity in a rodent model of schizophrenia. J. Psychopharmacol..

[21] Dejager L, Pinheiro I, Dejonckheere E, Libert C (2011). Cecal ligation and puncture: The gold standard model for polymicrobial sepsis?. Trends Microbiol..

[22] Lu YC, Yeh WC, Ohashi PS (2008). LPS/TLR4 signal transduction pathway. Cytokine.

[23] Remick DG, Newcomb DE, Bolgos GL, Call DR (2000). Comparison of the mortality and inflammatory response of two models of sepsis: Lipopolysaccharide vs. cecal ligation and puncture. Shock.

[24] de Castilho FM, Ribeiro ALP, Nobre V, Barros G, de Sousa MR (2018). Heart rate variability as predictor of mortality in sepsis: A systematic review. PloS One.

[25] Williams S (2022). Cardiac autonomic neuropathy in type 1 and 2 diabetes: Epidemiology, pathophysiology, and management. Clin. Ther..

[26] Iellamo F (2022). Complementary role of combined indirect and direct cardiac sympathetic (Hyper) activity assessment in patients with heart failure by spectral analysis of heart rate variability and nuclear imaging: Possible application in the evaluation of exercise training effects. J. Cardiovasc. Dev. Dis..

[27] Annane D (1999). Inappropriate sympathetic activation at onset of septic shock: A spectral analysis approach. Am. J. Respir. Crit. Care Med..

[28] Barnaby DP (2018). Use of the low-frequency/high-frequency ratio of heart rate variability to predict short-term deterioration in emergency department patients with sepsis. Emerg. Med. J. EMJ.

[29] Chen WL, Kuo CD (2007). Characteristics of heart rate variability can predict impending septic shock in emergency department patients with sepsis. Acad. Emerg. Med..

[30] Anderson TA (2017). Heart rate variability: Implications for perioperative anesthesia care. Curr. Opin. Anaesthesiol..

[31] Johnston JB (2001). Diminished adenosine A1 receptor expression on macrophages in brain and blood of patients with multiple sclerosis. Ann. Neurol..

[32] Castañé A, Soria G, Ledent C, Maldonado R, Valverde O (2006). Attenuation of nicotine-induced rewarding effects in A2A knockout mice. Neuropharmacology.

[33] Ma J (2021). Inhibition of cellular and animal inflammatory disease models by NF-κB inhibitor DHMEQ. Cells.

[34] Bairam A, Joseph V, Lajeunesse Y, Kinkead R (2009). Altered expression of adenosine A1 and A2A receptors in the carotid body and nucleus tractus solitarius of adult male and female rats following neonatal caffeine treatment. Brain Res..

[35] Nassar NN, Abdel-Rahman AA (2015). Brain stem adenosine receptors modulate centrally mediated hypotensive responses in conscious rats: A review. J. Adv. Res..

[36] Tseng CJ, Appalsamy M, Robertson D, Mosqueda-Garcia R (1993). Effects of nicotine on brain stem mechanisms of cardiovascular control. J. Pharmacol. Exp. Ther..

[37] Pavlov VA, Wang H, Czura CJ, Friedman SG, Tracey KJ (2003). The cholinergic anti-inflammatory pathway: A missing link in neuroimmunomodulation. Mol. Med..

[38] Zhai Q (2017). Selective activation of basal forebrain cholinergic neurons attenuates polymicrobial sepsis-induced inflammation via the cholinergic anti-inflammatory pathway. Crit. Care Med..

[39] Rogachev B (2006). Adenosine is upregulated during peritonitis and is involved in downregulation of inflammation. Kidney Int..

[40] Ramakers BP (2012). How systemic inflammation modulates adenosine metabolism and adenosine receptor expression in humans in vivo. Crit. Care Med..

[41] Bourhy L (2022). Neuro-inflammatory response and brain-peripheral crosstalk in sepsis and stroke. Front. Immunol..

[42] Barichello T, Generoso JS, Collodel A, Petronilho F, Dal-Pizzol F (2021). The blood-brain barrier dysfunction in sepsis. Tissue Barriers.

[43] Sonneville R (2013). Understanding brain dysfunction in sepsis. Ann. Intensive Care.

[44] Gong W, Wen H (1960). Sepsis induced by cecal ligation and puncture. Methods Mol. Biol..

[45] El-Mas MM, El-Gowelli HM, Ghazal AR, Harraz OF, Mohy El-Din MM (2009). Facilitation of central imidazoline I(1)-site/extracellular signal-regulated kinase/p38 mitogen-activated protein kinase signalling mediates the hypotensive effect of ethanol in rats with acute renal failure. Br. J. Pharmacol..

[46] El-Mas MM, Omar AG, Helmy MM, Mohy El-Din MM (2012). Crosstalk between central pathways of nitric oxide and carbon monoxide in the hypertensive action of cyclosporine. Neuropharmacology.

[47] El-Mas MM, Abdel-Rahman AA (1995). Upregulation of imidazoline receptors in the medulla oblongata accounts for the enhanced hypotensive effect of clonidine in aortic barodenervated rats. Brain Res..

[48] El-Mas MM, Abdel-Rahman AA (1999). Role of the sympathetic control of vascular resistance in ethanol-clonidine hemodynamic interaction in SHRs. J. Cardiovasc. Pharmacol..

[49] Omar AG, El-Mas MM (2004). Time-domain evaluation of cyclosporine interaction with hemodynamic variability in rats. Cardiovasc. Drugs Ther..

[50] Stein PK, Bosner MS, Kleiger RE, Conger BM (1994). Heart rate variability: A measure of cardiac autonomic tone. Am. Heart J..

[51] Berntson GG (1997). Heart rate variability: Origins, methods, and interpretive caveats. Psychophysiology.

[52] El-Naggar AE, El-Gowilly SM, Sharabi FM (2018). Possible ameliorative effect of ivabradine on the autonomic and left ventricular dysfunction induced by doxorubicin in male rats. J. Cardiovasc. Pharmacol..

[53] El-Mas MM, Abdel-Rahman AA (2013). Cardiovascular autonomic modulation by nitric oxide synthases accounts for the augmented enalapril-evoked hypotension in ethanol-fed female rats. Alcohol.

[54] Helmy MW, El-Gowelli HM, Ali RM, El-Mas MM (2015). Endothelin ETA receptor/lipid peroxides/COX-2/TGF-beta1 signalling underlies aggravated nephrotoxicity caused by cyclosporine plus indomethacin in rats. Br. J. Pharmacol..

[55] Chen GF, Sun Z (2006). Effects of chronic cold exposure on the endothelin system. J. Appl. Physiol..

[56] El-Mas MM, Abdel-Rahman AA (1997). Aortic barodenervation up-regulates alpha2-adrenoceptors in the nucleus tractus solitarius and rostral ventrolateral medulla: An autoradiographic study. Neuroscience.

[57] Ishioh M (2021). Activation of central adenosine A2B receptors mediate brain ghrelin-induced improvement of intestinal barrier function through the vagus nerve in rats. Exp. Neurol..

[58] Abuiessa SA, Helmy MM, El-Gowelli HM, El-Gowilly SM, El-Mas MM (2022). Dysregulated ACE/Ang II/Ang1-7 signaling provokes cardiovascular and inflammatory sequelae of endotoxemia in weaning preeclamptic rats. Eur. J. Pharmacol..

